# Prognostic impact of circulating tumor cell apoptosis and clusters in serial blood samples from patients with metastatic breast cancer in a prospective observational cohort

**DOI:** 10.1186/s12885-016-2406-y

**Published:** 2016-07-08

**Authors:** Sara Jansson, Pär-Ola Bendahl, Anna-Maria Larsson, Kristina E. Aaltonen, Lisa Rydén

**Affiliations:** Division of Oncology and Pathology, Department of Clinical Sciences Lund, Lund University, Medicon Village, SE-223 81 Lund, Sweden; Translational Cancer Research, Medicon Village, Lund University, SE-223 81 Lund, Sweden; Department of Surgery, Skåne University Hospital, SE-214 28 Malmö, Sweden; Department of Clinical Sciences Lund, Division of Surgery, Lund University, Medicon Village, SE-223 81 Lund, Sweden

**Keywords:** Circulating tumor cells, Metastatic breast cancer, Clusters, Apoptosis, Morphology

## Abstract

**Background:**

Presence of circulating tumor cells (CTCs) is a validated prognostic marker in metastatic breast cancer. Additional prognostic information may be obtained by morphologic characterization of CTCs. We explored whether apoptotic CTCs, CTC clusters and leukocytes attached to CTCs are associated with breast cancer subtype and prognosis at base-line (BL) and in follow-up (FU) blood samples in patients with metastatic breast cancer scheduled for first-line systemic treatment.

**Methods:**

Patients with a first metastatic breast cancer event were enrolled in a prospective observational study prior to therapy initiation and the CellSearch system (Janssen Diagnostics) was used for CTC enumeration and characterization. We enrolled patients (*N =* 52) with ≥5 CTC/7.5 ml blood at BL (median 45, range 5–668) and followed them with blood sampling for 6 months during therapy. CTCs were evaluated for apoptotic changes, CTC clusters (≥3 nuclei), and leukocytes associated with CTC (WBC-CTC, ≥1 CTC + ≥1 leukocytes) at all time-points by visual examination of the galleries generated by the CellTracks Analyzer.

**Results:**

At BL, patients with triple-negative and HER2-positive breast cancer had blood CTC clusters present more frequently than patients with hormone receptor-positive cancer (*P* = 0.010). No morphologic characteristics were associated with prognosis at BL, whereas patients with apoptotic CTCs or clusters in FU samples had worse prognosis compared to patients without these characteristics with respect to progression-free (PFS) and overall survival (OS) (log-rank test: *P* = 0.0012 or lower). Patients with apoptotic or clustered CTCs at any time-point had impaired prognosis in multivariable analyses adjusting for number of CTCs and other prognostic factors (apoptosis: HR_OS_ = 25, *P* < 0.001; cluster: HR_OS_ = 7.0, *P* = 0.006). The presence of WBC-CTCs was significantly associated with an inferior prognosis in terms of OS at 6 months in multivariable analysis.

**Conclusions:**

Patients with a continuous presence of apoptotic or clustered CTCs in FU samples after systemic therapy initiation had worse prognosis than patients without these CTC characteristics. In patients with ≥5 CTC/7.5 ml blood at BL, morphologic characterization of persistent CTCs could be an important prognostic marker during treatment, in addition to CTC enumeration alone.

Clinical Trials (NCT01322893), registration date 21 March 2011

**Electronic supplementary material:**

The online version of this article (doi:10.1186/s12885-016-2406-y) contains supplementary material, which is available to authorized users.

## Background

Hematogenous spread of cancer cells and subsequent formation of metastases in distant organs is the leading cause of death in cancer patients. A key step in metastasis is intravasation, i.e. the entrance of tumor cells into the hematologic or lymphatic system. Carcinoma-derived tumor cells circulating in the bloodstream, or circulating tumor cells (CTCs), in metastatic breast [1], prostate [2], colorectal [3], and lung [4, 5] cancer are associated with decreased progression-free survival (PFS) and overall survival (OS), and serial sampling after therapy initiation has also shown a prognostic importance of longitudinal CTC enumeration in metastatic breast cancer [1, 6–9].

Enumeration of CTCs in a liquid biopsy is a non-invasive monitoring that is easy to obtain via a peripheral blood sample and may hold promise for improving cancer prognostication and treatment. The most commonly used enrichment and detection technique for CTCs is the FDA approved CellSearch system (Janssen Diagnostics LLC, Raritan, NJ, USA). Molecular studies of CTCs are accumulating but few studies have thus far described morphological characteristics of CTCs, using either CellSearch-derived CTCs [10–14] or other methods for CTC isolation [15–20].

The malignant potential of CTCs has been suggested to be reflected in their morphological characteristics and these attributes are thus starting to be evaluated in clinical studies and related to outcome. A high fraction of apoptotic CTCs in the blood or apoptotic disseminated tumor cells (DTCs) in the bone-marrow in patients with solid tumors have been reported to be associated with decreased PFS and/or OS [4, 21–24]. The presence of CTC clusters has been reported for patients with metastatic colorectal, renal, prostate, lung and breast cancer [4, 12, 25–29] and the presence of clusters has been correlated to decreased survival in a few studies in small-cell lung cancer [4] and breast cancer [12, 14]. Diagnosis of CTC clusters (defined as ≥2 CTCs) have been related to poor outcome in stage III-IV breast cancer using the CellSearch system for CTC enumeration and characterization [14]. Paoletti et al [12] defined CTC clusters as ≥3 CTCs in the CellSearch gallery and for definition of apoptotic CTCs they applied M-30 staining as well as morphologic evaluation. They reported on prognostic information obtained by diagnosis of CTC clusters and apoptosis in metastatic triple-negative breast cancer showing that CTC clusters, but not apoptotic CTCs, added prognostic information in FU samples [12]. To date no consensus has been reached regarding the definitions of these morphologic characteristics using the CellSearch system and if additional biomarkers for diagnosis of apoptosis are needed.

Mixed clusters comprised of CTCs and leukocytes/white blood cells (WBC-CTC) have not been thoroughly investigated, but the complex relationship between CTCs and the immune system is gaining attention [30]. Generally, interactions between CTCs and the tumor microenvironment are still poorly understood but previous results have shown that specific immune cells have immunosuppressive properties in the peripheral blood, while this effect is absent in these cells in a tumor-associated environment [31, 32]. Also, association of CTCs with lymphocytes and platelets has been suggested to protect tumor cells against natural-killer (NK) cell-mediated lysis [33, 34].

We hypothesized that CTC clusters and apoptosis in metastatic breast cancer can provide prognostic information along CTC enumeration in all breast cancer subtypes and we sought to morphologically characterize CTCs in serial blood samples from patients with high risk (≥5 CTCs at base-line (BL)) metastatic breast cancer. All included patients were recently diagnosed with a first metastatic event and about to start first-line therapy in the metastatic setting. We explored whether apoptosis, CTC clusters and WBC-CTCs identified after CellSearch analysis without further staining were related to disease progression and survival, and if morphologic CTC characteristics differ among breast cancer subtypes and during follow-up (FU) from BL to 6 months after first-line systemic therapy. The present study shows that diagnosis of CTC clusters before start of systemic therapy correlate with an aggressive phenotype (triple-negative and HER2-subtype) and that presence of CTC clusters and apoptotic CTCs add prognostic information in FU samples even when adjusting for other prognostic factors.

## Methods

### Patients and study design

**Fig. 1:**
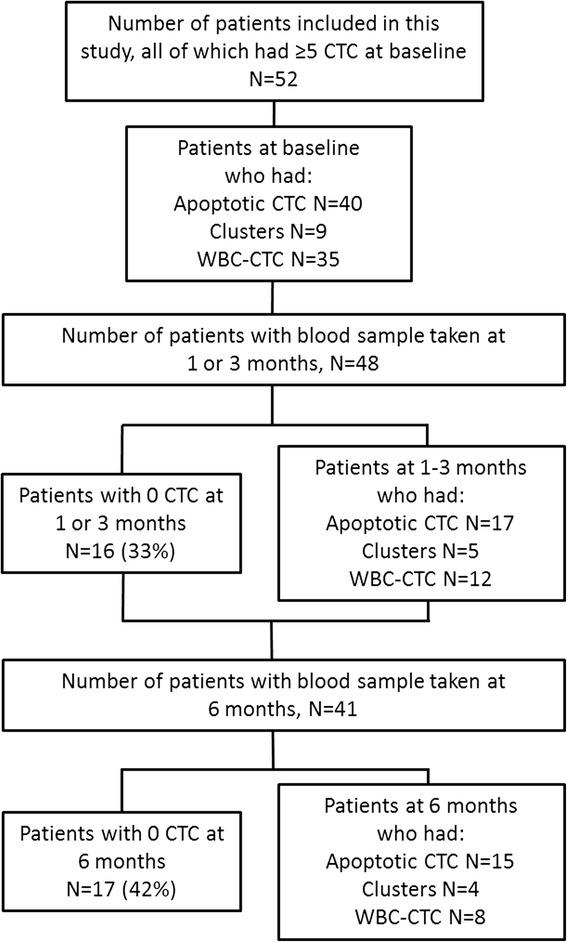
Flow-chart of CTC morphology study

An ongoing prospective monitoring trial at the Department of Oncology and Pathology, Lund University, Sweden aims to quantify and characterize CTCs in patients with metastatic breast cancer using progression-free survival (PFS) as a primary end-point. Women with distant metastases at diagnosis or first relapse metastatic breast cancer scheduled for first-line systemic treatment for metastatic disease in Lund, Malmö and Halmstad, have been included from 2011 (Clinical Trials NCT01322893) after oral and written informed consent (including publication of patient’s data). The study was approved by the Ethics committee at Lund University, Lund Sweden (LU 2010/135). Patient blood samples containing ≥5 CTCs at BL between 2011 and 2014 were analyzed in the present study. Patients were older than 18 years-of-age, with an ECOG performance status of ≤2 and a predicted life expectancy of >2 months. During the study, all patients received first-line systemic treatment for metastatic disease according to national guidelines (http://www.socialstyrelsen.se/publikationer2014/2014-4-2). Whole blood was collected from each patient at BL and after approximately 1, 3, 4, and 6 months of treatment or until disease progression. In the present study, we investigated the BL, 1–3 and 6 months blood samples (see Fig. 1). The 1 month sample was used only for 5 patients who lacked a 3-month sample (four of these patients were diseased before the scheduled 3-month sample and one sample was missing).

### CTC Analysis

**Fig. 2:**
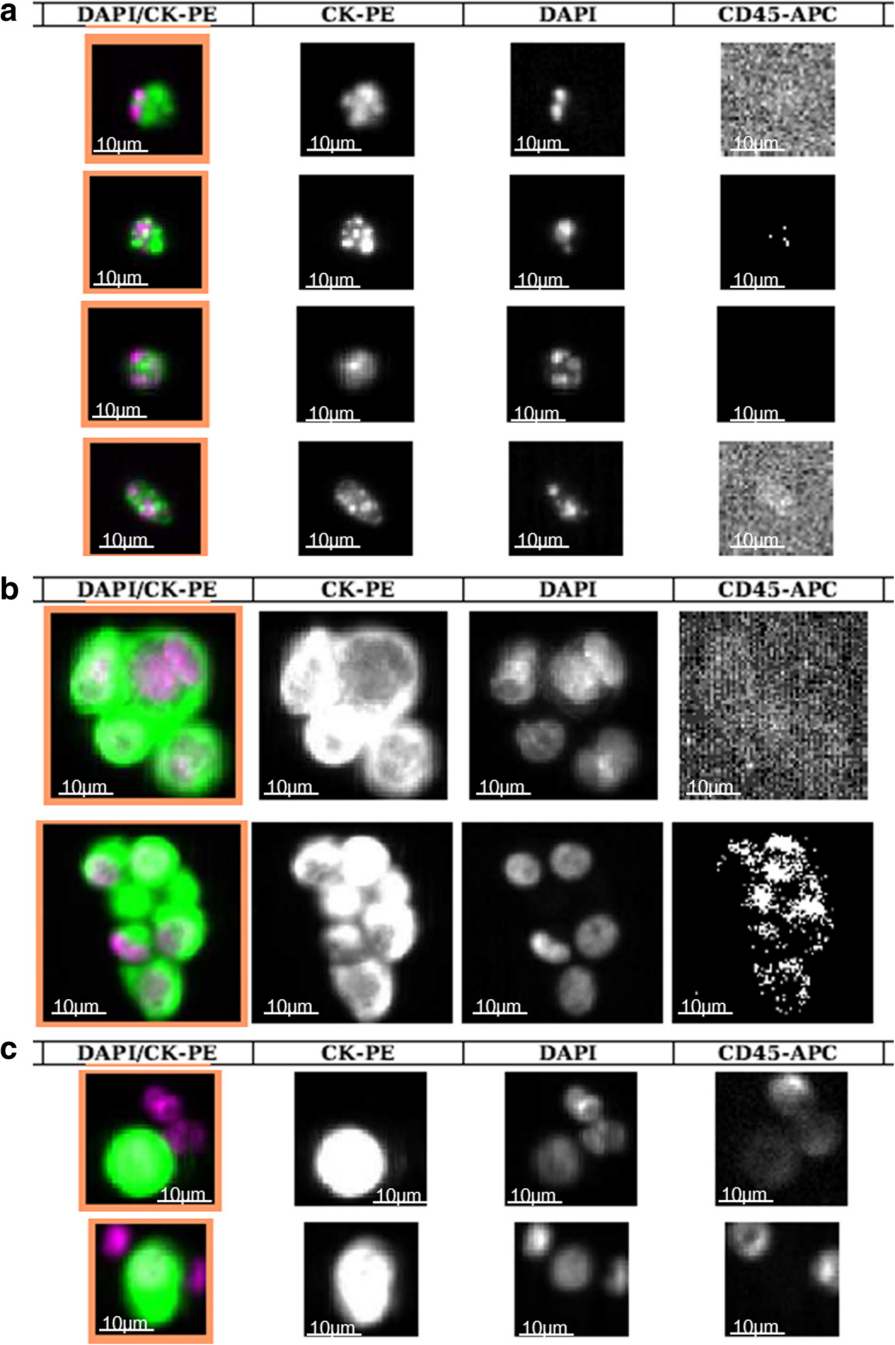
Photos of CTC morphology from CellTracks II Analyzer (10x). The four different columns depict from left to right: Nuclear DAPI staining (purple)/cytokeratin (CK)-PE (green) overlay, CK-staining, nuclear DAPI staining and CD45-APC staining. Scale bars have been added manually in each frame. **a** Examples of apoptotic CTCs from four different patients with characteristic fragmented and condensed apoptotic cell nuclei. **b** Examples of CTC clusters (defined as ≥3 nuclei). **c** Examples of WBC-CTCs

CTC detection and evaluation was performed using the CellSearch system (Janssen Diagnostics LLC, Raritan, NJ, USA) according to the manufacturer’s instruction. CellSearch is a semi-automated system that detects and enriches epithelial cells from whole blood (7.5 ml) using an epithelial cell adhesion molecule (EpCAM)-antibody coupled ferrofluid. All cells are counterstained with fluorescent antibodies against CD45 and cytokeratins (CK) 8, 18 and 19, and DAPI-stained for nuclear content, before scanning with a fluorescent microscope (CellTracks Analyzer II) to present them in a gallery for manual evaluation. CTCs are CK+/CD45-/DAPI+ cells fulfilling certain predefined criteria [35]. In this study, all gallery events were independently evaluated by two technicians trained and certified in the CellSearch technology. Events for which the assessment differed between the investigators were re-evaluated and a consensus was reached. Using the built in export function in the CellTracks Analyzer II system the cells selected as CTCs were grouped in a pdf gallery. Cells were subsequently assessed for apoptosis, CTC clusters and WBC-CTCs by two independent investigators (KA, SJ). Apoptotic cells were identified as cells with characteristic fragmented and condensed DAPI-stained nuclear morphology as defined by a clinical pathologist, and in the literature [36]. CTC clusters were defined as clusters of CTCs containing ≥3 distinct nuclei according to previous publications [12, 13]. By this definition it is less likely to incorrectly assign a mitotic CTC as a cluster. No additional staining of CTCs after CellSearch analysis was performed as this study aims to explore the feasibility of morphologic CTC characterization directly in the CellSearch gallery. This approach has previously been suggested in lung cancer [4]. WBC-CTCs were defined as ≥1 CTC clustered with ≥1 leukocyte and no definitive description of WBC-CTC has been published to date. Examples of apoptotic CTCs, CTC clusters, and WBC-CTCs are presented in Fig. 2a-c.

### Statistical analysis

Apoptotic CTCs, CTC clusters and WBC-CTCs were dichotomized into binary variables as previously described for CTC clusters [4, 12, 14] and apoptosis [4] and a patient was considered negative (no apoptotic CTC/CTC-cluster/WBC-CTC present) or positive (≥1 apoptotic CTC/CTC-cluster/WBC-CTC present).

Patient, tumor and CTC characteristics across subclasses of breast cancers and at different time-points were compared using a Pearson Chi-squared test or, if expected counts <5 in one or more of test cells, Fishers exact test. For ordinal variables with more than two categories, a linear-by-linear test for association was used and for variables measured on a continuous scale, the Mann-Whitney U-test was applied.

The primary end-point was PFS and the secondary end-point was overall survival (OS), both measured from BL to disease progression, death, or last FU. Survival data was retrieved from the patients’ medical charts and all events until March 2015 were recorded. Survival analyses of variables measured at 1–3 or 6 months was performed with landmark analysis for which PFS and OS were calculated from the time of sample taking, e.g. 1–3 or 6 months to disease progression, death, or last FU. Survival was evaluated using Kaplan-Meier (KM) analysis and log-rank test. Hazard ratios (HR) were calculated using Cox regression. Proportional hazards assumptions were checked graphically and with Schoenfeld’s test. Multivariable survival analyses were adjusted for the studied morphological variables, number of CTCs, breast cancer subgroup, age at diagnosis, time from first breast cancer diagnosis to diagnosis of metastasis [37], number and site of metastases. The presence of apoptotic CTCs or CTC clusters were also analyzed as time-dependent covariates using Cox regression models by splitting the FU time for each subject in the study into episodes during which both covariates were constant.

To account for the proportion of CTCs with the respective morphological characteristic, Cox regression was also done using the fraction of clustered/apoptotic/WBC associated CTCs per total number of CTCs in each patient. Statistical analyses were performed with IBM SPSS Statistics (version 22.0, IBM, Armonk, NY, USA) and STATA (version 13.1 StataCorp, (Stata Corp. College Station, TX, USA).

## Results

### Patient cohort and breast cancer subgroups

Table 1 offers patient characteristics and the study design is depicted in Fig. 1. Patients were divided into three subgroups based on hormone receptor status (estrogen receptor (ER) and progesterone receptor (PgR)) and HER2 (human epidermal growth factor receptor 2) status [38]. Breast cancer subtype was primarily derived from the primary tumor (*n* = 40) and secondly, if no primary tumor tissue was available, from metastases (*n* = 10). Two patients had insufficient tumor tissue for subtype assessment. The median FU for patients alive at the last review of the patient’s charts was as follows: 12 months (range 5–44) from BL samples, 10 months (range 1–42) from 1 to 3 month samples, and 15 months (range 1–38) from 6 month samples. Median PFS and OS from BL was 10 (95 % CI 9–16) and 19 (95 % CI 14–31) months, respectively. Total number of events until March 2015 in the cohort was 36 for PFS and 27 for OS.

**Table 1:** Patient and tumor characteristics in relation to breast cancer subtype^a^

Variables	All patients *N =* 52	Hormone receptor positive (ER+, PgR±, HER2-) *N =* 39	HER2 positive (HER2+, ER±, PgR±) *N =* 7	Triple-negative (ER-, PgR-, HER2-) *N =* 4	*P*-value
Age at MBC diagnosis					
Median (range)	60 (40–83)	64 (40–83)	57 (45–76)	51 (42–57)	0.10
< 50 years	12	9	1	2	0.45
≥ 50 years	40	30	6	2	
Time to recurrence					
Median (range in years)	5.3 (0–27.6)	5.1 (0–27.6)	1.7 (0–5.3)	1.3 (1.2–2.3)	0.007
Nr of patients with stage IV at diagnosis	9	5	4	0	0.44
NHG					
I	2	2	0	0	0.50
II	23	18	2	2	
III	18	13	2	2	
Unknown	9	6	3	0	
Ki67					
Low (≤20%)	5	5	0	0	0.071
High (>20%)	16	8	4	4	
Unknown	31	26	3	0	
First-line systemic therapy					
Endocrine only	11	11	0	0	^b^
Chemotherapy only	35	28	1	4	
HER2-directed (with chemotherapy)	6	0	6	0	
Metastatic site at BL					
Locoregional	3	1	0	2	0.30
Skeletal only	19	15	3	1	
CNS	1	1	0	0	
Visceral (two with unknown subtype)	28	21	4	1	
Other locations	1	1	0	0	
Number of metastatic locations					
1–2	32	24	4	3	0.74
3 or more	20	15	3	1	

^a^ Breast cancer subtype was derived from the primary tumor (*n* = 40) and, if no primary tumor tissue was available, from the metastasis (*n* = 10). Two patients had insufficient tissue for subtype assessment

^b^ No statistical analysis was performed for this clinically descriptive variable

WBC-CTC, white blood cells associated with CTC; ER, estrogen receptor; PgR, progesterone receptor; HER2, human epidermal growth factor receptor 2; BL, base-line; NHG, Nottingham histological grade; MBC, metastatic breast cancer; mo, months

### CTC counts

Median BL CTC counts did not differ among the three breast cancer subgroups (*P*-value = 0.32; Table 2). At 1–3 months, median CTC counts were greater in patients with triple-negative breast cancer (*P*-value = 0.007). This was not seen at 6 months, but fewer patients at this time point suggests caution for drawing conclusions from the results (*P*-value = 0.18; Table 2 and Fig. 1). Details on tumor, patient, and CTC characteristics in relation to breast cancer subgroup can be found in Tables 1 and 2.

**Table 2:** CTC counts and morphologic characteristics in relation to breast cancer subtype^a^

Variables	All patients *N =* 52	Hormone receptor positive (ER+, PgR±, HER2-) *N =* 39	HER2 positive (HER2+, ER±, PgR±) *N =* 7	Triple-negative (ER-, PgR-, HER2-) *N =* 4	*P*-value
CTC number					
CTC count at BL median (range)	45 (5–668)	44 (5–668)	111 (12–311)	88 (39–253)	0.32
CTC count at 1–3 mo median (range)	4 (0–263)	4 (0–263)	0 (0–9)	87 (75–144)	0.007
CTC count at 6 mo median (range)	1 (0–765)	1 (0–765)	0 (0–183)	2 (2–2)	0.18
≥ 5 CTC at 1–3 mo					0.29
Yes	19	15	1	3	
No	29	22	5	1	
Missing	4	2	1	0	
≥ 5 CTC at 6 mo					0.72
Yes	14	12	1	1	
No	27	19	5	1	
Missing	11	8	1	2	
Apoptosis					
Apoptotic CTC at BL					0.20
Yes	40	29	6	4	
Median number (range)	5 (1–54)	3 (1–52)	6.5 (1–54)	6.5 (5–40)	
Median fraction (range)	0.08 (0.01–0.33)	0.09 (0.01–0.33)	0.07 (0.04–0.17)	0.12 (0.05–0.18)	
No	12	10	1	0	
Missing	0	0	0	0	
Apoptotic CTC 1–3 mo					0.17
Yes	17	13	1	3	
Median number (range)	3 (1–18)	3 (1–18)	2	6 (3–9)	
Median fraction (range)	0.13 (0.01–1.0)	0.13 (0.01–1.0)	0.22	0.04 (0.04–0.10)	
No	31	24	5	0	
Missing	4	2	1	1	
Apoptotic CTCs 6 mo					0.49
Yes	15	12	1	2	
Median number (range)	2 (1–109)	2 (1–109)	2	3 (1–5)	
Median fraction (range)	0.09 (0.01–1.0)	0.11 (0.02–1.0)	0.01	0.29 (0.09–0.50)	
No	26	19	5	0	
Missing	11	8	1	2	
Clusters					
Clusters at BL					0.010
Yes	9	4	3	2	
Median number (range)	2 (1–18)	3 (1–18)	4 (1–4)	1.5 (1–2)	
Median fraction (range)	0.02 (0.003–0.03)	0.02 (0.003–0.03)	0.01 (0.005–0.02)	0.02 (0.02–0.03)	
No	43	35	4	2	
Missing	0	0	0	0	
Clusters at 1–3 mo					0.026
Yes	5	3	0	2	
Median number (range)	1 (1–4)	1 (1–4)		1 (1)	
Median fraction (range)	0.009 (0.006–0.02)	0.009 (0.006–0.02)		0.01 (0.007–0.01)	
No	43	34	6	1	
Missing	4	2	1	1	
Clusters at 6 mo					0.98
Yes	4	3	1	0	
Median number (range)	6 (1–16)	10 (2–16)	1		
Median fraction (range)	0.003 (0.001–0.006)	0.001 (0.001–0.005)	0.006		
No	37	28	5	2	
Missing	11	8	1	2	
WBC-CTC					
WBC-CTC at BL					0.45
Yes	35	26	6	3	
Median number (range)	4 (1–38)	3 (1–38)	6.5 (1–13)	4 (2–22)	
Median fraction (range)	0.05 (0.004–0.6)	0.05 (0.004–0.6)	0.07 (0.02–0.2)	0.04 (0.03–0.09)	
No	17	13	1	1	
Missing	0	0	0	0	
WBC-CTC at 1–3 mo					0.61
Yes	12	10	0	2	
Median number (range)	3.5 (1–101)	3.5 (1–28)		51.5 (2–101)	
Median fraction (range)	0.1 (0.02–1)	0.1 (0.04–1)		0.4 (0.02–0.7)	
No	36	27	6	1	
Missing	4	2	1	1	
WBC-CTC at 6 mo					0.49
Yes	8	6	1	1	
Median number (range)	6 (1–62)	5.5 (1–62)	9	3	
Median fraction (range)	0.05 (0.009–0.09)	0.04 (0.009–0.09)	0.05	0.05	
No	33	25	5	1	
Missing	11	8	1	2	

*WBC-CTC* white blood cells associated with CTC, *ER* estrogen receptor, *PgR* progesterone receptor, *HER2* human epidermal growth factor receptor 2, *BL* base-line, *NHG* Nottingham histological grade, *MBC* metastatic breast cancer, *mo* months

^a^ Breast cancer subtype was derived from the primary tumor (*n* = 40) and, if no primary tumor tissue was available, from the metastasis (*n* = 10). Two patients had insufficient tissue for subtype assessment

The established cut-off of ≥5 CTCs was investigated in survival analyses at 1–3 and 6 months. Data show significantly worse PFS and OS at both time-points for patients with ≥5 CTCs (Table 3 and Additional file 1). OS analysis at 1–3 months was also repeated without four patients with data from the 1 month sample due to patient deaths prior to 3-month sample acquisition and similar results were obtained. PFS and OS for each breast cancer subgroup for all time-points appear in Additional file 2. Results from multivariable analyses of CTC number appear in Table 3.

**Table 3:** Cox uni- and multivariable analysis by presence of apoptotic CTC, CTC clusters and WBC-CTC at base-line, 1–3 months, 6 months follow-up and by apoptotic CTC and clusters present at any time during the study (time-dependent covariates). At 1–3 and 6 months, CTC numbers categorized as ≥ 5 vs 0–4, is also presented

		PFS univariable			PFS multivariable			OS univariable			OS multivariable		
BL^a^		(*N =* 52)			(*N =* 50)^b^			(*N =* 52)			(*N =* 50)^b^		
		HR	95 % CI	*P*-value	HR	95 % CI	*P*-value	HR	95 % CI	*P*-value	HR	95 % CI	*P*-value
	Apoptosis	1.3	0.60–2.7	0.52	1.1	0.34–3.5	0.88	1.5	0.60–3.8	0.38	3.0	0.73–12	0.13
	Cluster	0.90	0.37–2.2	0.81	0.83	0.21–3.4	0.80	1.1	0.36–3.1	0.92	0.73	0.11–4.9	0.75
	WBC-CTC	1.0	0.50–2.0	0.98	0.82	0.33–2.0	0.67	0.76	0.35–1.7	0.49	0.69	0.20–2.3	0.54
1–3 months		(*N =* 45)			(*N =* 43)^c^			(*N =* 48)			(*N =* 46)^c^		
		HR	95 % CI	*P*-value	HR	95 % CI	*P*-value	HR	95 % CI	*P*-value	HR	95 % CI	*P*-value
	≥5 CTC	4.6	2.0–11	<0.001	4.3	1.1–17	0.041	5.8	2.3–14	<0.001	34	3.1–367	<0.001
	Apoptosis	4.9	2.1–11	<0.001	4.5	1.0–20	0.047	5.7	2.4–14	<0.001	10	1.2–87	0.031
	Cluster	7.4	2.3–24	0.001	2.0	0.30–14	0.46	9.8	2.5–38	0.001	3.1	0.48–20	0.23
	WBC-CTC	1.8	0.76–4.1	0.33	0.49	0.18–3.0	0.67	1.9	0.75–4.6	0.18	0.17	0.08–1.4	0.14
6 months		(*N =* 32)			(*N* = 30)^c^			(*N* = 42)			(*N* = 35)^c^		
		HR	95 % CI	*P*-value	HR	95 % CI	*P*-value	HR	95 % CI	*P*-value	HR	95 % CI	*P*-value
	≥ 5 CTC	6.1	2.1–17	0.001	53	4.5–613	0.002	2.7	1.0–7.1	0.047	0.47	0.02–13	0.66
	Apoptosis	4.8	1.7–13	0.003	2.1	0.34–13	0.42	6.2	2.2–18	0.001	79	5.5–1137	0.001
	Cluster	13	2.2–79	0.005	3.9	0.12–124	0.44	+∞^d^	Undefined	<0.001^e^	Not included		
	WBC-CTC	3.1	0.97–10	0.056	0.14	0.01–1.6	0.11	7.1	2.4–21	<0.001	23	1.1–458	0.042
Time-dependent		(*N* = 52)			(*N* = 50)^b^			(*N* = 52)			(*N* = 50)^b^		
		HR	95 % CI	*P*-value	HR	95 % CI	*P*-value	HR	95 % CI	*P*-value	HR	95 % CI	*P*-value
	Apoptosis	5.0	2.2–11	<0.001	6.7	2.4–19	<0.001	6.4	2.6–16	<0.001	25	5.2–115	<0.001
	Cluster	4.8	1.9–12	0.001	1.8	0.51–6.5	0.36	16	4.6–58	<0.001	7.0	1.7–28	0.006

*WBC-CTC* white blood cells associated with CTC, *PFS* progression free survival, *OS* overall survival, *HR* Hazard ratio calculated with Cox Regression, *CI* confidence interval

^a^ At BL, only patients with ≥5 CTCs were included and this variable (≥ 5 vs 0–4) is consequently not evaluated in survival analysis at this time point

^b^ Adjusted for: CTC number ≥20, breast cancer subgroup, age at diagnosis (continuous), time to recurrence, number (≥3 vs 1–2) and site of metastases (categorical on 5 levels)

^c^ Adjusted for: breast cancer subgroup, age at diagnosis, time to recurrence (continuous), number (≥3 vs 1–2) and site of metastases (categorical on 5 levels). Not adjusted for site of metastases at 6 months due to non-converging maximum likelihood estimation procedure

^d^ All four patients with clusters died before any of the patients in the group without clusters died (perfect prediction)

^e^ *P*-value from log-rank test

### Morphologic characteristics of CTCs in relation to CTC counts

All investigated CTC characteristics (apoptosis, clustering, WBC-CTCs) were significantly associated with CTC number at all time-points (*P*-value < 0.001; Additional file 3). No association to tumor burden as measured by the presence of visceral metastases was confirmed between either CTC characteristics or CTC number. At BL, a weak association existed between the presence of apoptotic CTCs and WBC-CTCs (*P*-value = 0.011) but not for the other investigated characteristics (Additional file 3). At 1–3 and 6 months, association among all investigated factors was high, likely due to many samples with 0 CTCs detected (16/48 patients at 1–3 months and 17/41 patients at 6 months; Additional file 3).

### Apoptotic CTCs

**Fig. 3:**
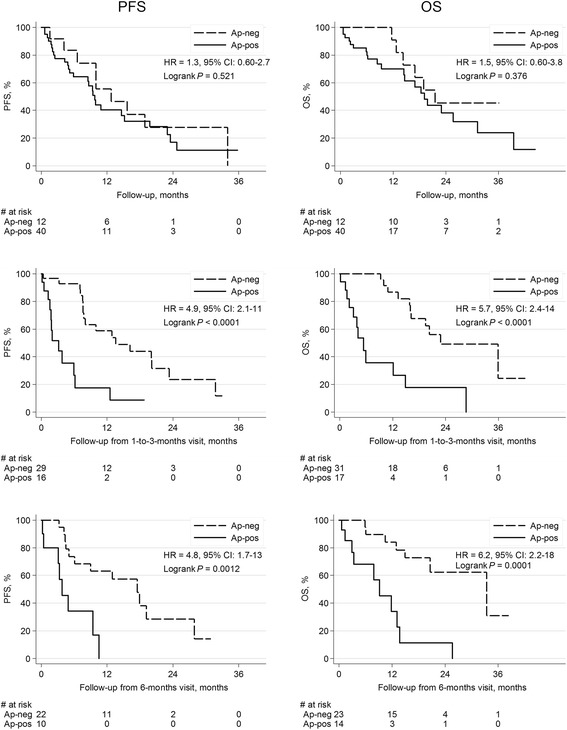
Kaplan-Meier survival plots and log-rank test by presence of apoptosis. Results from Cox-analyses are included in the respective graph. PFS and OS for patients with apoptotic CTCs present *vs* absent at BL, 1–3 and 6 months

CTC data appear in Table 2 and there was no difference in the number of patients with apoptotic CTCs among the three breast cancer subtypes at any time point (Table 2). The median number of apoptotic CTCs amongst patients positive for apoptosis at BL, 1–3 and 6 months were 5 (range 1–54), 3 (range 1–18) and 2 (range 1–109) respectively, and the corresponding fraction of apoptotic CTCs is depicted in Table 2. PFS or OS were not different for patients with or without apoptotic CTCs present at BL (Table 3 and Fig. 3). In contrast, at 1–3 months, significantly shorter PFS and OS were noted for patients with apoptotic CTCs present and this was also true at 6 months (Table 3 and Fig. 3). When adjusting for CTC number, breast cancer subgroup, age at diagnosis, time to recurrence, type and number of metastases, the presence of apoptotic CTCs was significantly related to increased HR at 1–3 and 6 months in terms of OS and at 1–3 months for PFS (Table 3). The fraction of apoptotic CTCs in relation to number of CTCs was not related to outcome (data not shown). Landmark analysis showed that patients with apoptotic CTCs present at any time-point during the study had significantly poorer PFS and OS compared to patients without apoptotic CTC. These results were consistent also in multivariable analysis (Table 3).

### CTC clusters

Fourteen patients (27%) had CTC clusters present at any time during the study and the median number of CTC clusters amongst patients positive for clusters at BL, 1–3 and 6 months were 2 (range 1–18), 1 (range 1–4) and 6 (range 1–16) respectively. Detailed information on all patients with CTC clusters appear in Additional file 4. At BL, CTC clusters were more frequently found in blood samples from patients with HER2-positive and triple-negative breast cancer compared to patients with hormone receptor-positive cancer (Table 2; *P-*value = 0.010). At 1–3 months, CTC clusters were still more frequent in the triple-negative breast cancer group (*P*-value = 0.026), whereas no significant difference could be found at 6 months (*P*-value = 0.98; Table 2). The fraction of CTC clusters in relation to CTC count is presented in Table 2.

**Fig. 4:**
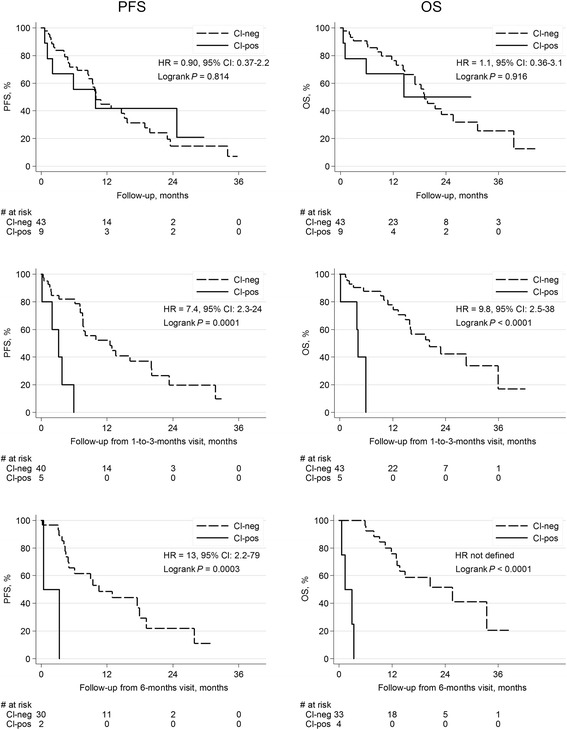
Kaplan-Meier survival plots and log-rank test by presence of CTC clusters. Results from Cox-analyses are included in the respective graph PFS and OS for patients with CTC clusters present *vs* absent at BL, 1–3 and 6 months

Survival of patients with CTC clusters present at BL was not different from patients without CTC clusters. At 1–3 months, shorter PFS and OS for patients with CTC clusters present in the blood were recognized compared to patients with no clusters present (Table 3 and Fig. 4). At 6 months, clusters were associated with shorter PFS whereas HR for OS was not defined because all patients in the cluster-positive group died prior to a patient death in the group without clusters (see Fig. 4 for Kaplan-Meier curves with log-rank *P*-value < 0.001). Multivariable analysis adjusting for CTC number and other prognostic factors, indicated increased HRs but no significant effect on prognosis when a patient was diagnosed with CTC clusters at 1–3 and 6 months (Table 3). Time-dependent landmark analysis confirmed that patients with clusters at any time during the study period had an increased risk of cancer progression and death compared to patients who never had CTC clusters (Table 3). The increased risk was also retained for OS in multivariable analysis (Table 3). In line with the inferior prognosis in patients with presence of CTC clusters in FU samples, patients with increasing fraction of CTC clusters per CTC number in FU samples had impaired prognosis (1–3 months: HR_PFS_ = 6.7, 95 % CI 2.4–18.7, *P <* 0.001; HR_OS_ = 12.1, 95 % CI 3.40–43.19, *P* < 0.001). The fraction of CTC clusters in 6 months FU samples was also significantly correlated to worse outcome, but due to the smaller sample size the results are uncertain.

### WBC-CTCs

Table 2 depicts patient WBC-CTC data and WBC-CTC presence did not differ among the three breast cancer subgroups at BL or at 1–3 or 6 months. The median number of WBC-CTC amongst patients positive for WBC-CTC at BL, 1–3 and 6 months were 4 (range 1–38), 3.5 (range 1–101) and 6 (range 1–62) respectively and the corresponding fraction of WBC-CTC is displayed in Table 2. No significant difference in survival was observed for patients with WBC-CTCs present at BL or 1–3 months compared to patients with no WBC-CTCs. However, at 6 months, worse survival in terms of PFS and OS was observed for patients with WBC-CTC (Table 3 and Additional file 5). In contrast, multivariable analysis indicated that the presence of WBC-CTC had a positive effect on survival for both PFS and OS at 1–3 months and on PFS at 6 months, but these results were not significant (Table 3). At 6 months the presence of WBC-CTC was significantly related to worse OS in multivariable analysis (Table 3). The fraction of WBC-CTC per number of CTC did not add any prognostic information (data not shown).

## Discussion

The prognostic information of CTC enumeration in FU blood samples has been shown in a number of studies [1, 6–9] but the added value of CTC characterization in FU samples is largely unknown. Apoptotic CTCs and CTC clusters in metastatic breast cancer has gained recent attention and in the present exploratory study we investigated the significance of these morphologic characteristics using the CellSearch gallery in a homogenous cohort from patients with poor prognosis (≥5 CTCs at base-line (BL)) metastatic breast cancer undergoing first-line systemic therapy including all breast cancer subtypes. We show that the presence of apoptotic CTCs and CTC clusters in FU blood samples at 1–3 and 6 months after treatment initiation indicated poorer prognosis. Moreover, Cox-models with time-dependent covariates confirmed that the presence of apoptotic CTCs and CTC clusters at any time-point during the study was associated with increased mortality independent of other prognostic factors such as CTC numbers and breast cancer subtype.

Our findings agree with a recent study of metastatic triple-negative breast cancer in which presence of CTC clusters diagnosed using the CellSearch gallery in FU blood samples during the first month of treatment was associated to significantly worse PFS [12]. However, we included patients with all subtypes of breast cancer with CTC number ≥5 at BL and limited inclusion of patients to those about to start first-line systemic therapy, thus the prognostic information yielded by morphologic characteristics is not only related to pretreated patients or any specific subtype of breast cancer. Another recent publication has also suggested that detection of CTC clusters is important when evaluating prognosis in breast cancer [14]. Both publications [12, 14] used the CellSearch system for CTC enumeration and characterization, but applied separate definitions for CTC clusters. In the study by Mu et al*.* [14], including patients with breast cancer stage III and IV, the presence of CTC clusters (defined as ≥2 CTCs) at BL was associated to worse prognosis in terms of decreased PFS. A majority of patients (69 out of 115) in this study had inflammatory breast cancer and the authors conclude that these patients had larger clusters; five of seven patients in this study with CTC clusters of ≥3 CTCs had inflammatory breast cancer. The publication by Paoletti et al*.* [12] investigated the importance of CTC clusters and apoptosis in metastatic triple-negative breast cancer. They found that CTC clusters (defined as ≥3 CTCs) but not apoptotic CTCs (defined by any M-30 staining and/or visual characteristics of apoptosis) in FU blood samples during treatment was associated with worse PFS.

In the present study retained CTC clusters added significant prognostic information after 1–3 and 6 months of first line therapy. Also, clusters were found significantly more often in patients with triple-negative breast cancer compared to hormone receptor-positive cancer. Previous studies indicate that CTCs within clusters may represent a more malignant and mesenchymal subpopulation of tumor cells [20, 28]. Early experiments in animal models indicate that intravenously injected tumor cell clusters have a greater tendency to form metastases than an equal number of injected single CTCs [39, 40]. Adding to these findings, Aceto et al*.* recently reported that CTC clusters in breast cancer have a 23- to 50-fold increased metastatic potential [20]. Furthermore, no apoptotic CTCs were found within CTC clusters in the blood of patients with lung cancer, suggesting that tumor cells in clusters have a survival advantage compared to solitary CTC [13]. We confirm these results in breast cancer, as no apoptotic CTCs were found in cell clusters in our patients. Likely, clustered CTCs evade anoikis by retaining cell-to-cell survival signals by expressing proteins responsible for intracellular junctions, exemplified by plakoglobin [20]. Moreover, characterization of CTCs within clusters supports the hypothesis that CTCs within clusters are different from single CTC—they are non-proliferating as indicated by the absence of Ki67 expression [4].

In this cohort of patients with metastatic breast cancer, we found no apoptotic CTCs within clusters, but all patients with clusters also had apoptotic single CTCs. The presence of apoptotic CTCs was also related to poor outcome at 1–3 and 6 months, whereas no association to prognosis could be seen at BL. Persistent apoptotic CTCs over time indicates a failure to respond to systemic therapy with retained proliferation and cell turnover in the metastatic lesion and/or primary tumor [4]. This could be a possible explanation for the dismal prognosis for patients with retained apoptotic CTCs during treatment as found in the present study. Our results are not consistent with previous studies using CellSearch for detection and analysis of apoptotic CTCs [4, 12]. In contrast to our data, Hou et al*.* found that presence of ≥1 apoptotic CTC at BL was associated with significantly worse PFS and OS in lung cancer [4] (the study did not analyze apoptotic CTCs in FU samples). On the other hand, in metastatic triple-negative breast cancer, Paoletti et al*.* found no prognostic effect of apoptotic CTCs either at BL or in FU samples at day 15 and 29 [12]. Our data suggest that samples taken after several cycles of systemic treatment have a higher significance for prognostic information. In contrast to the studies above, we included no further staining (e.g. M30) after CellSearch analysis and only used morphological criteria for diagnosis of apoptotic CTCs. It is possible that early stage apoptotic CTCs, as detected by additional staining, carry less prognostic information. Also, we found no evidence for prognostic significance by analyzing the fraction of apoptotic CTCs as applied by Paoletti et al. [12].

The prognostic importance of WBC-CTCs in patients with metastatic breast cancer has, to our knowledge, not been previously investigated. Interestingly, univariable analysis in this cohort indicated worse prognosis for patients with WBC-CTCs present whereas adjustment for other prognostic factors such as CTC number, age, and time to recurrence lowered HR to less than 1, suggesting that WBC-CTCs could be favorable for survival. Possibly further characterization of CTC-associated leukocytes may provide prognostic information [41], but the CellSearch methodology only specifies leukocyte presence by the phenotype CK-/CD45+/DAPI+. Thus, no detailed information on leukocyte types associated with CTC is available within the present study.

As enumeration of CTCs was prognostic in the presented cohort and we found an association between CTC number and all morphologic characteristics, we also accounted for the fraction of CTCs with the respective morphological characteristic in relation to the total CTC count in each patient. The fraction of CTC clusters added prognostic information in FU samples, supporting that CTC clusters add important prognostic information to enumeration of CTCs. The fraction of apoptotic CTCs and WBC-CTCs was not significantly related to prognosis in contrast to the presence of the respective morphological trait.

A limitation of the study is that we included only 52 patients with metastatic breast cancer in this exploratory analysis and the statistical power was consequently limited. Although we selected patients based on cut-point for CTC enumeration, the median CTC count at BL was 45 for all included patients as an indication of a group of patients with dismal prognosis. Another limitation of the study is that patients within triple-negative and HER2-positive subgroups were underrepresented but diagnosed significantly more often with CTC clusters at BL. Four patients with HER2-positive subtype were included, three of which cleared their CTCs after 6 months of FU and one with progressive disease after 5 months (Additional file 4). The patient with disease progression had increased number of CTCs and also presented with clusters at 6 months. The most efficient eradication of CTCs and CTC clusters occurred in a patient treated with HER2-directed double blockade diagnosed with 311 CTC at BL and four clusters. The finding indicates that dynamic changes of CTCs evolving under the pressure of systemic therapy may be predictive for treatment success. In contrast, all patients in the difficult to treat triple-negative subgroup (*N =* 4) had a constant presence of apoptotic CTCs during the study (Table 2), both at BL and during chemotherapy. A higher cell turn-over rate in patients with triple-negative subtype may be one explanation for the dismal prognosis in this breast cancer subtype.

This exploratory study was performed by evaluating CTCs captured with the FDA approved CellSearch system without any downstream staining and our study can be repeated in more patients in clinical studies using CTCs as surrogate marker. CTC apoptosis, clusters and WBC-CTCs were assessed morphologically directly in the CellSearch gallery following assessment guidelines proposed in previous publications. Even if the CTC galleries provided by the CellTracks Analyzer II system only offers pictures with 10 x magnifications, CellSearch is the most used and well documented CTC isolation system available. Thus, being able to extract putative prognostic information by including basic and easy-to-assess morphologic characteristics in addition to today’s enumeration, an even more powerful prognostic tool may lie ahead for patients presenting with ≥5 CTC/7.5 ml blood at BL. Confirmation that apoptotic CTCs and CTC clusters observed in CellSearch analyses are not a result of artifacts but indeed true morphologic characteristics have been presented previously [4, 12, 42]. We applied a published definition of a CTC cluster to enable comparison with previous studies [12, 13] and the definition of apoptosis was according to criteria used in clinical pathology [13, 36] without the need of further staining after CellSearch analysis. Further validation of morphological assessment of CTCs in independent and larger cohorts is warranted.

## Conclusions

The clinical value of monitoring CTC counts in metastatic breast cancer has recently been confirmed in a large meta-analysis [1]. Morphologic characterization of CTCs by assessment of apoptotic CTCs and CTC clusters may offer additional prognostic information to enumeration in patients with ≥5 CTC/7.5 ml blood at BL.In the present study, we evaluated apoptotic CTCs, CTC clusters and WBC-CTCs in patients with poor prognosis metastatic breast cancer using the standardized CTC capture and presentation system from Janssen Diagnostics, the CellSearch system. Serial sampling from patients treated systemically with first-line approaches was performed from therapy initiation to 6 months. We observed significantly worse prognosis for patients with apoptotic CTCs and CTC clusters present in peripheral blood during treatment, suggesting that morphologic characterization of persistent CTCs during treatment may be an important prognostic marker, in addition to CTC enumeration alone.

## Abbreviations

BL, base-line; CI, confidence interval; CK, cytokeratin; CTC, circulating tumor cell; EpCAM, epithelial cell adhesion molecule; ER, estrogen receptor; FU, follow-up; HER2, human epidermal growth factor receptor 2; HR, Hazard ratio; KM, Kaplan-Meier; OS, overall survival; PFS, progression-free survival; PgR, progesterone receptor; WBC, white blood cell, leukocyte

## Additional files

Additional file 1: Survival analysis using KM plots (log-rank *P*-value) and Cox analysis for patient with CTC number 0–4 vs ≥ 5 at 1–3 and 6 months (at BL, only patients with ≥ 5 CTC were included). PFS and OS were investigated as endpoints. (TIF 895 kb) Additional file 2: Survival analysis using KM plots (log-rank *P*-value) and Cox analysis by breast cancer subgroup. PFS and OS were investigated as endpoints. (TIF 975 kb) Additional file 3: Association between CTC characteristics at base-line, 1–3 and 6 months. (PDF 458 kb) Additional file 4: Summary of characteristics for patients with clusters at either BL, 1–3 or 6 months (*n* = 14). (PDF 265 kb) Additional file 5: Survival analysis using KM plots (log-rank *P*-value) and Cox analysis for patients with WBC-CTC present vs absent at BL, 1–3 and 6 months. PFS and OS were investigated as endpoints. (TIF 1304 kb) Additional file 6: Supporting data. (XLS 73 kb)
