# Supplementary figures and images for: Prognostic impact of circulating tumor cell apoptosis and clusters in serial blood samples from patients with metastatic breast cancer in a prospective observational cohort
Source: BMC Cancer. 2016 Jul 8;16:433. doi: 10.1186/s12885-016-2406-y (PMC4938919; doi:10.1186/s12885-016-2406-y)

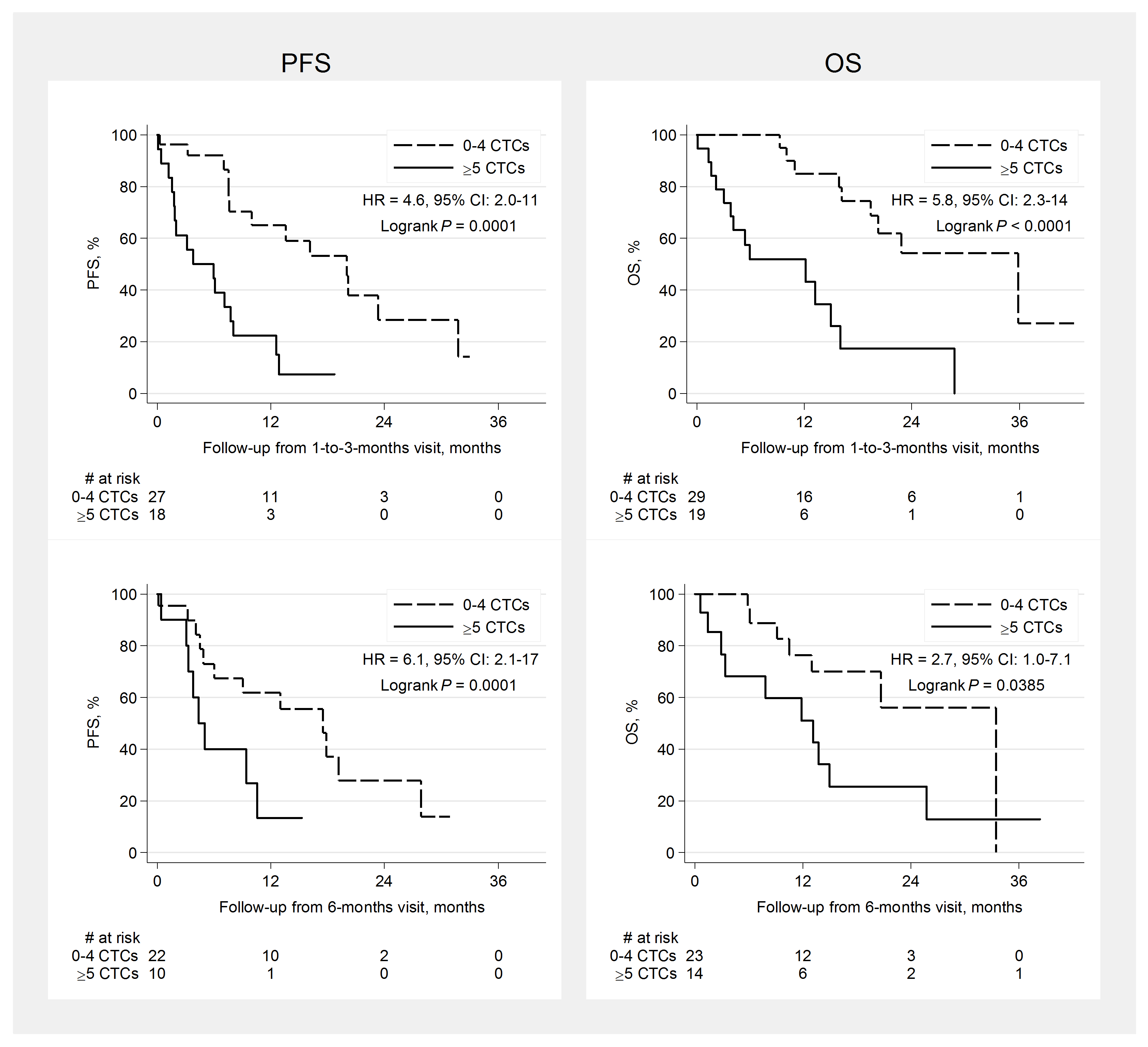

Supplement: Additional file 1: — Survival analysis using KM plots (log-rank P-value) and Cox analysis for patient with CTC number 0–4 vs ≥ 5 at 1–3 and 6 months (at BL, only patients with ≥ 5 CTC were included). PFS and OS were investigated as endpoints. (TIF 895 kb) [file 12885_2016_2406_MOESM1_ESM.tif]

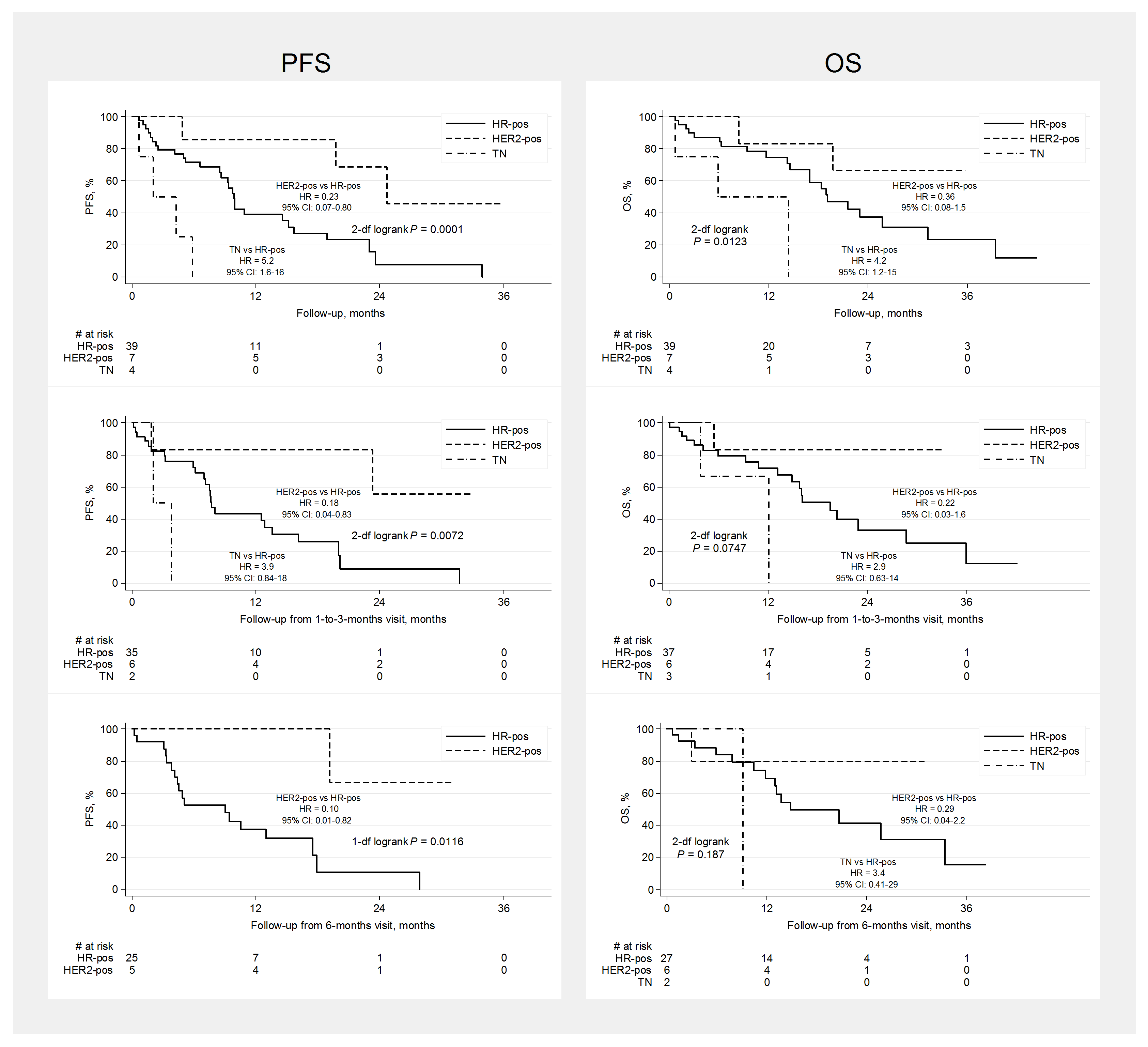

Supplement: Additional file 2: — Survival analysis using KM plots (log-rank P-value) and Cox analysis by breast cancer subgroup. PFS and OS were investigated as endpoints. (TIF 975 kb) [file 12885_2016_2406_MOESM2_ESM.tif]

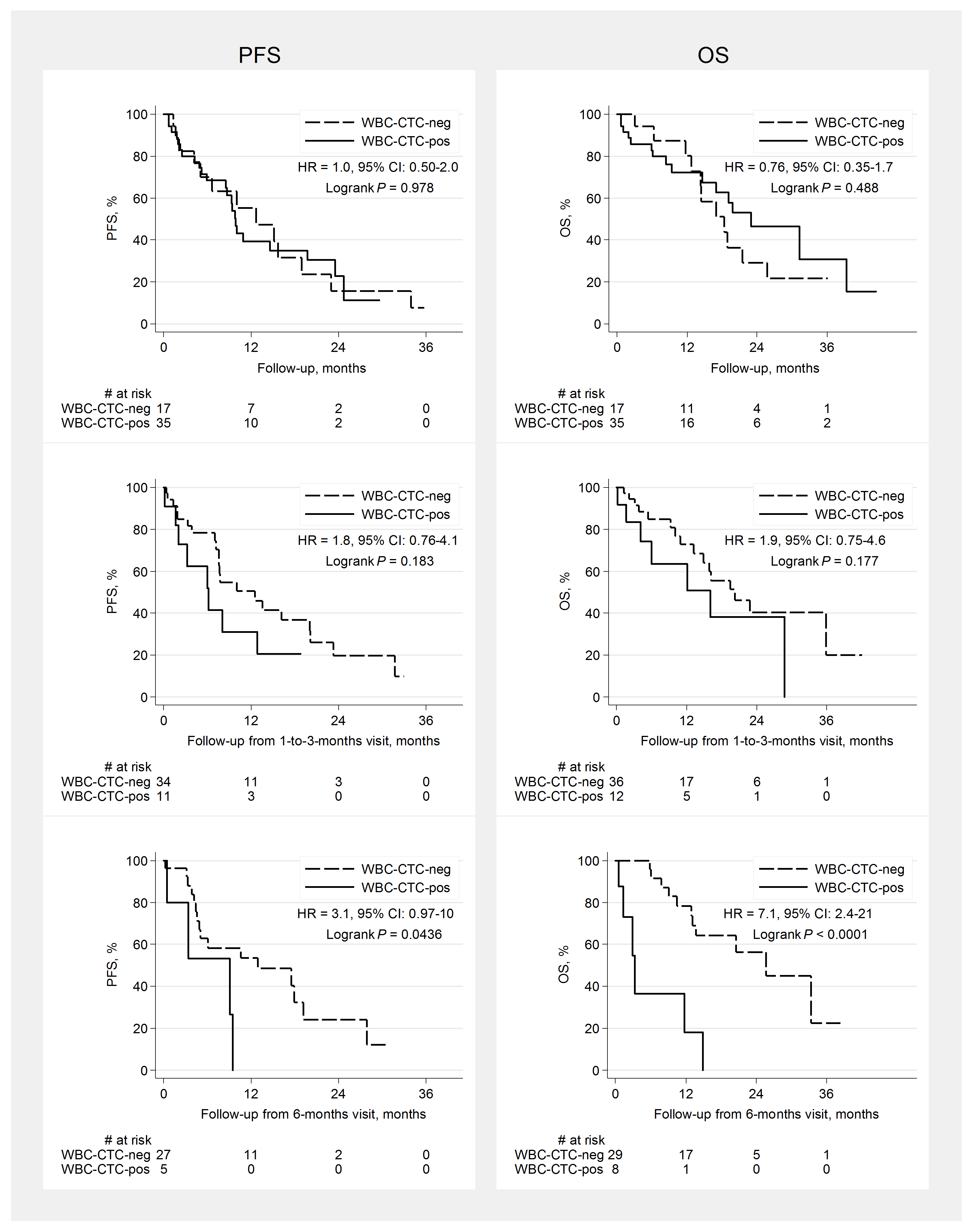

Supplement: Additional file 5: — Survival analysis using KM plots (log-rank P-value) and Cox analysis for patients with WBC-CTC present vs absent at BL, 1–3 and 6 months. PFS and OS were investigated as endpoints. (TIF 1304 kb) [file 12885_2016_2406_MOESM5_ESM.tif]
