# Supplementary material for: Prognostic impact of circulating tumor cell apoptosis and clusters in serial blood samples from patients with metastatic breast cancer in a prospective observational cohort
Source: BMC Cancer. 2016 Jul 8;16:433. doi: 10.1186/s12885-016-2406-y (PMC4938919; doi:10.1186/s12885-016-2406-y)
Supplement: Additional file 3: — Association between CTC characteristics at base-line, 1–3 and 6 months. (PDF 458 kb) [file 12885_2016_2406_MOESM3_ESM.pdf]

Additional file 3. Association between CTC characteristics at base-line, 1-3 and 6 months

**Base-line**			**Apoptosis**		**Cluster**		**WBC-CTC**		**Visceral met**		**Stage IV at diagnosis**		**Number of CTC**
			Yes	No	Yes	No	Yes	No	Yes	No	Yes	No	Median
			40	12	9	43	35	17	21	31	9	43	45
**Apoptosis**	Yes	40			9[a]	31	31	9	15	25	8	32	80
	No	12	-		0	12	4	8	6	6	1	11	11
					P = 0.097[b]		***P* = 0.011**[b]		*P* = 0.51[b]		*P* = 0.67[b]		***P* < 0.001**[c]
**Cluster**	Yes	9					8	1	3	6	4	5	173
	No	43			-		27	16	18	25	5	38	35
							P = 0.24[b]		*P* = 0.72[b]		***P* = 0.037**[b]		***P* < 0.001**[c]
**WBC-CTC**	Yes	35							14	21	8	27	86
	No	17					-		7	10	1	16	12
									P = 1.0[d]		*P* = 0.24[b]		***P* < 0.001**[c]
**Visceral met**	Yes	21									3	18	43
	No	31							-		6	25	55
											P = 0.72[b]		*P* = 0.67[c]
**Stage IV at diagnosis**	Yes	9											111
	No	43									-		38
													***P* = 0.023**[c]
**Number of CTC**	Median	45											-

1-3 months			Apoptosis		Cluster		WBC-CTC		Visceral met		Stage IV at diagnosis		Number of CTC
			Yes	No	Yes	No	Yes	No	Yes	No	Yes	No	Median
			17	31	5	43	12	36	17	31	7	41	4
**Apoptosis**	Yes	17			5[a]	12	8	9	5	12	2	15	58
	No	31	-		0	31	4	27	12	19	5	26	0
	Missing	4			***P* = 0.004**[b]		***P* = 0.015**[b]		*P* = 0.52[d]		*P* = 1.00[b]		***P* < 0.001**[c]
**Cluster**	Yes	5					4	1	2	3	0	5	144
	No	43			-		8	35	15	28	7	36	1
	Missing	4					***P* = 0.011**[b]		*P* = 1.00[b]		*P* = 1.00[b]		***P* = 0.001**[c]
**WBC-CTC**	Yes	12							2	10	2	10	68
	No	36					-		15	21	5	31	1
	Missing	4							*P* = 0.17[b]		*P* = 1.00[b]		***P* < 0.001**[c]
**Visceral met**	Yes	17									1	16	1
	No	31							-		6	25	4
	Missing	4									*P* = 0.40[b]		*P* = 0.70[c]
**Stage IV at diagnosis**	Yes	7											1
	No	41									-		4
	Missing	4											*P* = 0.20[c]
**Number of CTC**	Median	4											-

6 months			Apoptosis		Cluster		WBC-CTC		Visceral met		Stage IV at diagnosis		Number of CTC
			Yes	No	Yes	No	Yes	No	Yes	No	Yes	No	Median
			15	26	4	37	8	33	14	27	7	34	1
**Apoptosis**	Yes	15			4	11	7	8	4	11	2	13	48
	No	26	-		0	26	1	25	10	16	5	21	0
	Missing	11			***P* = 0.013**[b]		***P* = 0.002**[b]		*P* = 0.44[d]		*P* = 1.00[b]		***P* < 0.001**[c]
**Cluster**	Yes	4					4	0	3	1	0	4	440
	No	37			-		4	33	11	26	7	30	1
	Missing	11					***P* = 0.001**[b]		*P* = 0.11[b]		*P* = 1.00[b]		***P* = 0.001**[c]
**WBC-CTC**	Yes	8							3	5	1	7	153
	No	33					-		11	22	6	27	0
	Missing	11							*P* = 1.00[b]		*P* = 1.00[b]		***P* < 0.001**[c]
**Visceral met**	Yes	14									1	13	1
	No	27							-		6	21	1
	Missing	11									*P* = 0.39[b]		*P* = 0.86[c]
**Stage IV at diagnosis**	Yes	7											0
	No	34									-		1
	Missing	11											*P* = 0.11[c]
**Number of CTC**	Median	1											-

[a] Apoptotic CTC were only found as single cells, not within clusters

[b] Fishers exact test

[c] Mann-Whitney U-test

[d] Pearson Chi Square test

WBC-CTC, white blood cells associated with CTC; met, metastasis
