# Supplementary material for: Prognostic impact of circulating tumor cell apoptosis and clusters in serial blood samples from patients with metastatic breast cancer in a prospective observational cohort
Source: BMC Cancer. 2016 Jul 8;16:433. doi: 10.1186/s12885-016-2406-y (PMC4938919; doi:10.1186/s12885-016-2406-y)
Supplement: Additional file 4: — Summary of characteristics for patients with clusters at either BL, 1–3 or 6 months (n = 14). (PDF 265 kb) [file 12885_2016_2406_MOESM4_ESM.pdf]

Additional file 4. Summary of characteristics for patients with clusters at either BL, 1-3 or 6 months (n=14)

Patient no	Subtype	Time point	Nr of CTCs	Nr of clusters	Apoptotic CTC present	WBC-CTC present	Patient status	PFS (months)	First line treatment
1	HR+	BL	384	8	Yes	Yes	D	2	Chemotherapy[a]
		1 mo	263	4	Yes	Yes			
		6 mo	N/A D	N/A D	N/A D	N/A D			
2	HR+	BL	668	18	Yes	Yes	D	1	Chemotherapy[a]
		3 mo	N/A D	N/A D	N/A D	N/A D			
		6 mo	N/A D	N/A D	N/A D	N/A D			
3	HR+	BL	153	5	Yes	Yes	PD	12	Chemotherapy[a]
		3 mo	15	0	No	No			
		6 mo	2	0	No	No			
4	HR+	BL	151	1	Yes	Yes	SD	22	Hormonal therapy
		3 mo	5	0	Yes	Yes			
		6 mo	0	0	No	No			
5	HR+	BL	86	0	Yes	Yes	D	6	Chemotherapy[c]
		3 mo	111	1	Yes	Yes			
		6 mo	695	16	Yes	Yes			
6	HR+	BL	82	0	Yes	Yes	D	11	Hormonal therapy
		3 mo	181	1	Yes	Yes			
		6 mo	184	2	Yes	Yes			
7	HR+	BL	9	0	Yes	No	D	6	Chemotherapy[d]
		3 mo	31	0	Yes	No			
		6 mo	765	1	Yes	Yes			
8	HER2+	BL	311	4	Yes	Yes	PD	25	HER2-directed therapy[e]

		3 mo	0	0	No	No			
		6 mo	0	0	No	No			
9	HER2+	BL	189	1	Yes	Yes	SD	8	HER2-directed therapy[f]
		3 mo	0	0	No	No			
		6 mo	0	0	No	No			
10	HER2+	BL	173	4	Yes	Yes	SD	31	HER2-directed therapy[f]
		3 mo	0	0	No	No			
		6 mo	0	0	No	No			
11	HER2+	BL	38	0	Yes	Yes	D	5	HER2-directed therapy[f]
		3 mo	9	0	Yes	No			
		6 mo	183	1	Yes	Yes			
12	TN	BL	104	2	Yes	Yes	D	7	Chemotherapy[b]
		3 mo	75	1	Yes	No			
		6 mo	N/A W	N/A W	N/A W	N/A W			
13	TN	BL	39	1	Yes	No	D	11	Chemotherapy[b]
		3 mo	87	0	Yes	Yes			
		6 mo	2	0	Yes	No			
14	TN	BL	71	0	Yes	Yes	PD	5	Chemotherapy[b]
		3 mo	144	1	Yes	Yes			
		6 mo	57	0	Yes	Yes			

Foot-note to treatment regimen:

[a] Poly-chemotherapy with anthracycline (FEC)

[b] Platinum containing regimen (carboplatinum) + gemcitabine

[c] Weekly paclitaxel single therapy

[d] Oral chemotherapy (capecitabine)

[e] Her-2-directed therapy – single blockade (trastuzumab) + chemotherapy
[f] Her-2-directed therapy – double blockade (trastuzumab + pertuzumab) + chemotherapy
HR+, hormone receptor positive; HER2+, human epidermal growth factor receptor 2 positive; TN, triple negative; BL, baseline; mo, months; N/A D, not available patient diseased; WBC-CTC, white blood cells associated with CTC; N/A W, not available patient withdrew from the study; D, diseased; PD, progressive disease; SD, stable disease.
